# Motherhood and decision-making among women living with HIV in developed countries: a systematic review with qualitative research synthesis

**DOI:** 10.1186/s12978-021-01197-6

**Published:** 2021-07-10

**Authors:** Ariadna Huertas-Zurriaga, Patrick A. Palmieri, Joan E. Edwards, Sandra K. Cesario, Sergio Alonso-Fernandez, Lidia Pardell-Dominguez, Karen A. Dominguez-Cancino, Juan M. Leyva-Moral

**Affiliations:** 1 Hospital Universitari Germans Trias I Pujol, Badalona, 08916 Barcelona, Spain; 2 Grupo de Investigación Enfermera en Vulnerabilidad Y Salud (GRIVIS), Universitat Autònoma de Barcelona, Avda. Can Domènech, Edifici M. Despatx M3/213, Bellaterra (Cerdanyola del Vallès), 08193 Barcelona, Spain; 3 South American Center for Qualitative Research, Universidad Norbert Wiener, Av. Arequipa 444, Torre 2, Piso 4, Lima, 15046 Perú; 4 College of Graduate Health Studies, A. T. Still University, 800 W. Jefferson Street, Kirksville, MO 63501 USA; 5 Center for Global Nursing, Texas Woman’s University, 6700 Fannin St, Houston, TX 77030 USA; 6 EBHC South America: A JBI Affiliated Group, Calle Cartavio 406, Suite 402, Lima, 15023 Peru; 7 Nelda C. Stark College of Nursing, Texas Woman’s University, 6700 Fannin St, Houston, TX 77030 USA; 8 Recerca i Innovació en Cures Infermeres, Hospital Universitari Germans Trias I Pujol, Badalona, 08916 Barcelona, Spain; 9 Department D’Infermeria, Facultat de Medicina, Universitat Autònoma de Barcelona, Avda. Can Domènech, Edifici M. Despatx M3/213, Bellaterra (Cerdanyola del Vallès), 08193 Barcelona, Spain; 10 Escuela de Enfermería, Universidad Científica del Sur, Carr. Panamericana Sur 19, Villa EL Salvador, Lima, 15067 Perú; 11 Escuela de Salud Pública, Universidad de Chile, Independencia 939, Independencia, 8380453 Santiago de Chile, Chile

**Keywords:** AIDS, HIV, Decision-making, Pregnancy, Reproductive health, Women, SIDA, VIH, Toma de decisiones, Embarazo, Salud reproductiva, Mujeres

## Abstract

**Background:**

Women living with HIV (WLH) lack evidence-based information about reproductive options while managing pressures from family, clinicians, and communities to give up the idea of having children. As the reproduction intentions of WLH are not well understood, stigmatizing behaviors force them to hide their disease to avoid rejection by their family, partner, and social networks. Compliance with social norms, fear of stigma, and discrimination influence their experience. Current research is individual qualitative studies lacking the synthesis perspective necessary to guide intervention development. The purpose of this study was to synthesize the evidence to explain the reproductive decision-making process for WLH in developed countries.

**Methods:**

A systematic review with qualitative research synthesis was conducted through searches in 10 electronic databases (CINAHL, EMBASE, MEDLINE, Scopus, Social Science Citation Index, Web of Science, Google Scholar, Cuidatge, Cuiden Enfispo, and SciELO). Studies published in journals from 1995 to 2019 with qualitative data about reproductive decision-making among WLH in developed countries were eligible for inclusion. Developed country was operationalized by membership in the OECD for comparative conditions of social wellbeing and economic stability. The CASP and JBI checklists for qualitative research were used to assess study quality and methodological integrity. Thematic analysis and qualitative meta-summary techniques were used for the synthesis.

**Results:**

Twenty studies from 12 developed countries were included in the synthesis. Findings were organized into 3 meta-themes from 15 themes and 45 subthemes, including: (1) Shattered identity, (2) Barriers, inequities, and misinformation, (3) Coping, resiliency, and support. Reproductive decision-making was perceived as a complex process influenced by facilitators and barriers. The facilitators helped WLH cope with their new situation to become more resilient, while the barriers made their situation more difficult to manage.

**Conclusion:**

WLH encounter reproductive decision-making with knowledge deficits and limited social support. An integrated approach to holistic care with comprehensive multidisciplinary counseling is needed to support WLH. Clinicians could benefit from professional development to learn how to be authentically present for WLH, including engaging in conversations, demonstrating compassion, and understanding situations. Evidence-based clinical practice guidelines need to be tailored for the family planning and sexual health needs of WLH.

## Resumen

**Antecedentes:**

Las mujeres que viven con el VIH (MVV) carecen de información basada en evidencias sobre las opciones reproductivas mientras son presionadas por la familia, los profesionales de la salud y los miembros de la comunidad para renunciar a la idea de tener hijos. Como las intenciones reproductivas de las MVV no son comprendidas, las conductas estigmatizantes las obligan a ocultar su enfermedad para evitar el rechazo de su familia, pareja y grupos sociales. El cumplimiento de las normas sociales, el miedo al estigma y la discriminación influyen en su experiencia. La presente investigación está compuesta por estudios cualitativos que de forma individual carecen de la perspectiva de síntesis necesaria para guiar el desarrollo de las intervenciones. El propósito de este estudio fue sintetizar la evidencia para explicar el proceso de toma de decisiones reproductivas para las MVV en los países desarrollados.

**Métodos:**

Se realizó una revisión sistemática con síntesis de investigación cualitativa mediante búsquedas en 10 bases de datos electrónicas (CINAHL, EMBASE, MEDLINE, Scopus, Social Science Citation Index, Web of Science, Google Scholar, Cuidatge, Cuiden Enfispo y SciELO). Los estudios publicados en revistas de entre 1995 y 2019 que contuvieran datos cualitativos sobre la toma de decisiones reproductivas entre las MVV en países desarrollados fueron elegibles para su inclusión. Se consideraron países desarrollados aquellos que pertenecieran a la OCDE con el objetivo de comparar condiciones de bienestar social y estabilidad económica. Las listas de verificación CASP y JBI para la investigación cualitativa se utilizaron para evaluar la calidad del estudio y la integridad metodológica. Para la síntesis se utilizaron técnicas de análisis temático y metanálisis cualitativo.

**Resultados:**

En la síntesis se incluyeron veinte estudios de 12 países desarrollados. Los hallazgos se organizaron en 3 metatemas de 15 temas y 45 subtemas, incluyendo: (1) Identidad fragmentada, (2) Barreras, inequidades y desinformación, (3) Afrontamiento, resiliencia y apoyo. La toma de decisiones reproductivas se percibió como un proceso complejo influenciado por factores facilitadores y barrera. Los facilitadores ayudaron a las MVV a afrontar su nueva realidad para volverse más resilientes, mientras que las barreras hicieron que su situación fuera más difícil de manejar.

**Conclusión:**

Las MVV enfrentan la toma de decisiones reproductivas con déficits de conocimiento y apoyo social limitado. Es necesario adoptar un enfoque holístico de atención integral con asesoramiento multidisciplinario para acompañar a las MVV. Los clínicos podrían beneficiarse del desarrollo profesional para aprender a estar verdaderamente presentes para las MVV, participando en reflexiones, demostrando compasión y comprendiendo sus situaciones. Las guías de práctica clínica basadas en la evidencia deben adaptarse a las necesidades de planificación familiar y salud sexual y reproductiva de las MVV.

Plain Language Summary

Women living with HIV can become pregnant and deliver a healthy baby due to advances in medicine. Being a mother is an important role that gives meaning to life for most women. For women living with HIV thinking about having a baby is difficult because HIV complicates many areas of daily living. When women living with HIV try to speak with physicians and nurses about having a baby, they often do not feel supported and report feeling stigmatized. This review of the scientific literature summarizes the experiences of women living with HIV in developed countries as they considered having a baby. Ten electronic databases were searched for studies published between 1995 and 2019 reporting interviews with women living with HIV about becoming pregnant and having a baby. From the 4519 articles identified, 20 were included for review with 1395 participants from 12 developed countries. After abstracting and analyzing the interviews, three themes were developed to summarize the process described by women living with HIV as they considered pregnancy and the possibility of having a baby, including: (1) Shattered identity, (2) Barriers, inequities, and misinformation, and (3) Coping, resiliency, and support. When women living with HIV consider having a baby, they need to feel comfortable and safe speaking with physicians and nurses about family planning. They also need more support from their partner, as well as family and friends. Strategies need to be implemented to improve the family planning process for women living with HIV, including education health care providers about speaking to women about pregnancy and having a healthy baby.

## Introduction

Globally there are nearly 40 million people living with HIV, more than half are women [1]. In 2018, 82% of pregnant women living with HIV (WLH) had access to antiretroviral treatment to prevent mother-to-child transmission, with higher rates in developed countries [2]. The wider treatment coverage and improved adherence among WLH has rapidly reduced AIDS-related deaths among women [3]. Increased access to highly active anti-retroviral therapy (HAART) shifted HIV from an acute illness to a chronic disease [4] for women in terms of life expectancy, quality of life, and the opportunity for motherhood [5–7]

Motherhood is an important role that gives meaning to life for many women; however, decisions about pregnancy and childbearing are often complex as HIV impacts many aspects of daily living [8]. The factors influencing reproductive decision-making include concerns about the baby being born with HIV and the longevity of life for the mother [9, 10]. Pregnancy with HIV brings increased risk for complications [5, 8] including maternal infection and adverse perinatal outcomes [9, 11, 12]. Following the introduction of HAART, however, mother-to-child transmission was reduced to less than 1% in developed countries [13]. The knowledge transfer of this evidence into clinical practice, within communities, and throughout the general public has been slow [14].

Although cesarean section was the recommended mode for delivery prior to HAART [15, 16] European guidelines now promote vaginal delivery [17] as maternal viral load was identified as the most significant risk factor for perinatal HIV infection. Despite favorable conditions for a minimal risk pregnancy in developed countries, researchers suggest HIV serostatus and knowledge about HIV transmission does not significantly influence reproductive decision-making for WLH [18, 19]. Due to the available conception and contraception options, WLH can avoid pregnancy or safely attempt pregnancy with good outcomes [20]. Yet, only a third of WLH in developed countries reported a pregnancy since their diagnosis [11]. Despite significant advancements in medical technology and improvements in clinical practice, reproductive decision-making continues to be guided by historical perspectives and outdated evidence.

### Rational for review

WLH lack evidence-based information about reproductive options while managing pressures from family, clinicians, and communities to give up the idea of having children [19, 21]. Since reproduction intentions of WLH are not well understood [22], stigmatizing behaviors force women to hide their disease in order to avoid rejection by their family, partner, and social networks [8]. Compliance with social norms, fear of stigma, and discrimination influence their experiences and guide their behaviors [23]

Misinformation and prejudice continue in clinical practice [24] as WLH do not feel supported by their clinicians. They report discrimination and stigma in some cases [25]; with the resulting fear and anxiety negatively impacting their relationships with clinicians and their confidence in the health system [26]. WLH require comprehensive evidence-based care to safely fulfill their decisions about motherhood, whether this involves preventing [9] or safely advancing a pregnancy [27].

Compared to developed countries, longstanding variations in human rights, socioeconomic conditions, public policies, and health systems in developing countries differentially restrict women from accessing quality health services, achieving sexual health, and making reproductive decisions [28–31], especially women infected with HIV [32–42]. In developing countries, WLH have less favorable and more varied conditions for sexual health and reproductive services than WLH in developed countries [43–46] due to a complex mixture of gender and disease associated inequities related to cultural norms [47], religious doctrines [48], poverty [49], health policy [50], health services [51], and emergency conditions [52]. Developed countries provide WLH with the most supportive environments for sexual health and to engage in reproductive decision-making. As such, the purpose of this study was to explain the reproductive decision-making process of WLH in developed countries according to their lived experiences through a synthesis of the qualitative literature. The study fills an important gap in the literature as the research is individual qualitative studies lacking the synthesis perspective necessary to guide intervention development [14, 53].

## Methods

Qualitative research synthesis (QRS) [54], or the aggregative approach to synthesis [55], is aligned with the philosophy of pragmatism where “meanings exist as ready-made” [56] to convey “practical usefulness” [57] that informs decisions and practices at the clinical or policy levels [58]. As such, the result of a QRS is more than a simple summary of findings from the literature [59]. For this reason, we completed a QRS with a multinational team approach [60] to interpret data from systematically selected articles to generate new insights and additional knowledge [61]. The research protocol (CRD42018091971) was registered with the International Prospective Register of Systematic Reviews, PROSPERO [62]. The results are reported according to the Preferred Reporting Items for Systematic Reviews and Meta-Analyses (PRISMA) statement [63], and the Enhancing Transparency in Reporting the synthesis of Qualitative research (ENTREQ) recommendation [64].

### Inclusion criteria

Studies published in peer-reviewed journals between January 1995 (the year HAART appeared) and December 2019 using qualitative methods to address reproductive decision-making in WLH were included in this study. The term ‘developed country’ was operationalized by membership in the Organization for Economic Co-operation and Development (OECD). In this regard, the 35 OECD [65] member countries are substantially similar in their compliance with international laws, adherence to human rights conventions, and adoption of evidence-based clinical practice guidelines for HIV/AIDS [66–69]. The publication language was limited to English, French, German, Portuguese, and Spanish as these were the language proficiencies of the research team. Studies were excluded if the target population was women less than 18 years or more than 50 years of age, women living in prisons or psychiatric institutions, or mixed-method studies or systematic reviews in which qualitative data could not be separated from the quantitative.

### Search strategy

Multiple electronic databases were searched including CINAHL, EMBASE, MEDLINE (through PubMed), Scopus, Social Science Citation Index, Web of Science, Google Scholar, and the Spanish databases Cuidatge, Cuiden Enfispo, and SciELO from January 1995 to December 2019. Although most qualitative research articles for this review were anticipated to be indexed in the MEDLINE database [70], all research articles related to reproductive decision-making and WLH were of interest. As such, the database filters specific to research methods were not used to limit the possibility for bias in article selection [71]. To minimize unintended bias in the search strategy, published recommendations were referenced for searching databases, including CINAHL [72], EMBASE [73], MEDLINE [74] and PsycINFO [75].

Keywords, defined by the research team and informed by systematic reviews in related areas, were joined by Boolean operators for the database searches. Keywords were used to avoid problems with the unique thesaurus terms for each database with varied meanings between disciplines [73, 76]. The keywords included: HIV, AIDS, human immunodeficiency virus, acquired immunodeficiency syndrome, women, women living with HIV, childbearing, desire for children, fertility, family planning, pregnancy, reproduction, reproductive behavior, reproductive choice, reproductive health, decisions, decision making, intention, and mother-to-child transmission.

The search strategies were tested in November 2017. An example of the algorithm used for the PubMed search strategy is provided in Table 1. The full searches were completed the week of December 17, 2018 with an updated search for the calendar year 2019 completed in June 2020. This systematic review serves as the foundation for a living systematic review [77] with updates scheduled every five years.

**Table 1:** Search strategy for PubMed

("Reproductive Health" [Mesh]) AND women living with HIV
("Decision Making" [Mesh]) AND "Fertility" [Mesh]) AND "HIV" [Mesh]
("Reproductive Behavior" [Mesh]) AND "Fertility" [Mesh]) AND "HIV" [Mesh]
("Reproductive Behavior" [Mesh]) AND "Decision Making" [Mesh]) AND "HIV" [Mesh]
("Reproductive Behavior" [Mesh]) AND "HIV" [Mesh]
("Fertility" [Mesh]) AND "Intention" [Mesh]) AND "HIV" [Mesh]
("Reproduction" [Mesh]) AND "Decision Making" [Mesh]) AND "HIV" [Mesh]
("Decision Making" [Mesh]) AND "Reproductive Health" [Mesh]) AND "HIV" [Mesh]
("Family Planning Services" [Mesh]) AND "HIV" [Mesh]
Childbearing decision AND HIV
Childbearing decision AND AIDS
Reproductive AND decision making AND HIV
Reproductive AND decision making AND AIDS
Fertility intention AND HIV
Fertility intention AND AIDS
Fertility AND decision making AND HIV
Fertility AND decision making AND AIDS
Child desire AND AIDS
Child desire AND HIV
Reproductive choice AND women living with HIV

### Study screening

**Figure 1.**
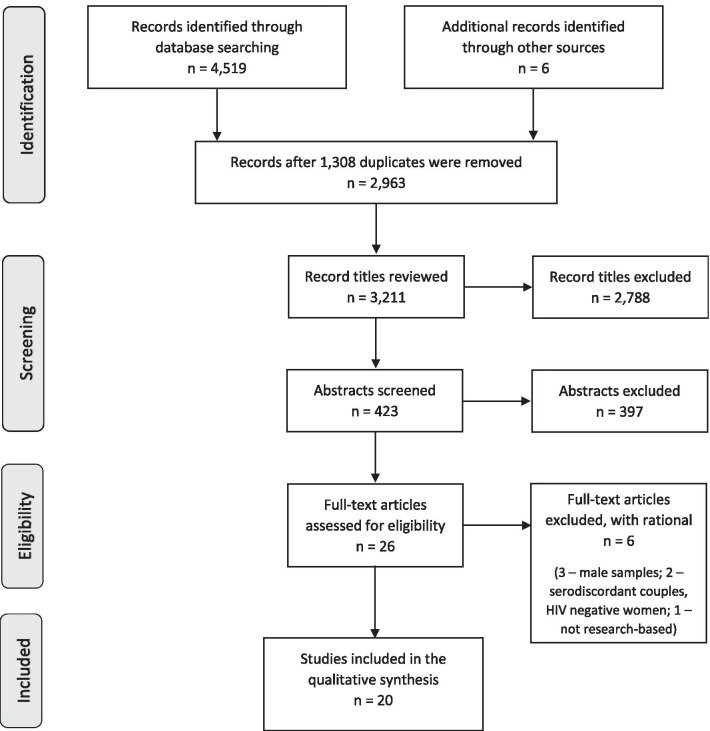
PRIMSA diagram

The study screening was completed with a structured process [78]; first by title review, then by abstract review, and finally by full text review by four pairs of experienced reviewers. The study titles were first independently screened by paired reviewers to identify the studies that met the inclusion criteria. Next, the reviewers evaluated the abstracts for inclusion, reviewing a different group than the previous step. Finally, the reviewers assessed the full text of the remaining articles by strictly applying the inclusion and exclusion criteria. During each round, a third reviewer checked the work of each review pair for errors. If there was disagreement between the paired reviewers, the document advanced to the next review process to limit deselection bias. The primary investigator checked the level of agreement between review pairs for the title and abstract phases, with the goal of 95% agreement. During the full text review, the excluded and included articles were checked by another pair of reviewers as a final verification. At this point, six studies were excluded from the synthesis due to samples with all men (n = 3), serodiscordant couples with HIV negative women (n = 2), and no participants due to a descriptive rather than research-based article (n = 1). The PRISMA diagram provides an overview of the screening process (see Fig. 1).

### Quality assessment

The research team included the appropriate methods and content experts [79] to develop a clear, justified, and focused review question [80] with realistically defined objectives [81] for an expansive search strategy to identify relevant research [82]. Four pairs of reviewers independently screened the articles by applying the inclusion criteria and assessing the risk of bias. Discrepancies were solved by AHZ and JLM; and then reviewed by an external researcher. Each article included in the full text analysis was peer-reviewed (AHZ and JLM), and independently assessed (AHZ, JLM, and PAP) for methodological criteria with the 10-item Critical Appraisal Skills Programme (CASP) qualitative checklist [83]. Consensus was required for inclusion of an article. Congruence between theory, methods, and analysis, researcher reflexivity, and ethical protections was also assessed with the JBI Critical Appraisal Checklist for Qualitative Research Studies [55]. The checklists were applied to assess the minimum quality of study methods, adequacy of ethical protections, and trustworthiness [84] rather than to exclude interview data from papers as this could introduce bias [85]

### Data extraction

The primary focus for the data extraction was the results and the conclusions sections of each article. General data extracted from the articles, line-by-line, were assembled in an Excel spreadsheet, including study setting, aim, participants, study design, direct quotes, and key findings. Then, the data were imported into Nvivo® to digitally record the codes that emerged as themes from the coding [86]. The level of coding was words, phrases, and sentences.

### Data synthesis

Following the recommendations of Sandelowski and Barroso [54], the data analysis was circular rather than linear to facilitate movement between the emergent themes from the findings [87]. This process resulted in a taxonomy of findings from sustained comparisons, concepts translated in vivo, and imported concepts [88]. The taxonomy was comprised of items with different semantic relations, either within the same or between different themes.

In a staged process [89], two researchers (AHZ and JLM) conducted an inductive analysis [90] followed by a deductive process. The results were discussed and triangulated with two other researchers (JEE and SKC) and checked by additional researchers (PAP and KDC). The relationships, similarities, and dissonances were synthesized across studies. During this process, key concepts and conceptual themes were identified. Relevant findings from one study were compared to the themes, metaphors, and concepts in the other studies. As recommended by King [91], the thematic analysis was not finalized until all the data were reviewed and the codes checked multiple times.

### Trustworthiness

The protocol for this QRS was designed to maximize descriptive validity (factual accuracy of data), interpretive validity (validity referred to in descriptions of member checking), theoretical validity (credibility of researcher interpretations), and pragmatic validity (utility and transferability of knowledge) [87, 92, 93]. In this regard, several techniques were incorporated into the protocol such as the check and rechecks of the initial search results, maintenance of an audit trail, multiple researchers completing article appraisals, expert peer reviews, team 'huddles' to discuss individual and comparative appraisals, member checks, and ongoing discussion and negotiation [94–97].

### Ethics

Ethics approval was not necessary as the searches were conducted in public databases. There were no human subjects involved in this study. However, the included studies were evaluated for ethical standards with the JBI Checklist. Each of these studies was approved by an ethics committee or institutional review board.

## Results

A total of 4,519 articles were identified through the systematic literature searches and reduced to a sample of 20 qualitative articles for analysis (see Table 2). Each article met the requirement for inclusion using the CASP checklist. However, one article [98] was assessed as weak in theoretical and methodological congruence with the JBI checklist but included to limit selection bias. The articles represented 1,395 participants with data from 12 of the 35 OECD member countries; about half the studies were from urban areas within the United States.

**Table 2:** Summary of included studies

Author/s Year	Sample/setting	Methodology and methods	Main findings
Alvarez-del Arco (2019)	20 WLH, between 18 and 45 years of age (Spain)	Qualitative study, design not stated. Data collection: Interviews (open ended). Dates: May and July 2013. Participants resided in Spain but represented Eastern Europe (4), Latin America (6), Spain (9), and Sub-Saharan African (1)	The findings were presented as three topics impact of HIV diagnosis, concept of motherhood, and dimensions of motherhood with four dimensions, including motherhood ideal, desire for procreation, the decision of motherhood, experience (or the lack of experience) of motherhood. The authors noted some dimensions emerged from the interview data as well as organized with the theoretical model
Barnes and Murphy (2009)	80 WLH, childbearing age, living in Oakland and Chicago (United States)	Grounded theory. Data collection: Interviews (semi-structured). Dates: 1995 to 2000	WLH reproductive decisions are based on their judgment of the relative weight of positive aspects of motherhood versus the often-negative pressures of social and public opinion
Barnes (2013)	36 WLH, mothers, from Oakland and Rochester (United States)	Grounded theory. Data collection: Interviews (in-depth). Dates: 2005 to 2009	WLH who had living children experienced longevity from fulfilling dreams of seeing their children grow up despite the unique challenges from their HIV status. The longevity offered possibilities for regaining contact with children who had been given up for adoption, were or had been in foster care, or lived with family members. WLH felt living longer offered more possibilities of becoming a mother with pregnancy, but opportunities were complicated with reconciling past reproductive experiences and poor choices
Campero et al. (2010)	20 WLH heterosexual and 20 men > 18 years old in four states of Mexico	Grounded theory. Data collection: Interviews (in-depth). Dates: 2003 to 2004	Limited support and counseling is a barrier to exercising sexual and reproductive rights of participants, especially women. Principal issues included feeling frustrated and confused, fear of re-infection, limited information, lack of power to negotiate condom use, social stigma and discrimination, and limited access to services and technologies
Carlsson-Lalloo et al. (2016)	18 qualitative studies with a total of 588 WLH interviewed from wealthier countries outside the Asian and African continents	Meta-ethnography. Data collection: Interview and observational data. Dates: 1997 to 2012. Locations: USA (11), Canada (2), UK (2), Australia (1), Ireland (1), and Brazil (1). Two systematic searches (sexuality and reproduction) in CINAHL and MEDLINE. Articles assessed with Critical Appraisal Skills Programme	HIV infection is a burden in relation to sexuality and reproduction. The weight of the burden can be heavier or lighter. Conditions making the HIV burden heavier included: HIV as a barrier, feelings of fear and loss, whereas motherhood, spiritual beliefs, and supportive relationships make the HIV burden lighter
Cuca and Rose (2016)	20 WLH, > 18 years old, diagnosed at least 1 year prior to study; pregnant at least once since their HIV diagnosis living in San Francisco (United States)	Grounded theory with situational analysis. Data collection: Interviews (in-depth) and observations. Dates: 2009, October to 2010, February; and 2012, October and 2013, February	Reproductive choices are made in situations of chaos, instability, and stigmatization. For some women, providers are sources of stigma. Participants demonstrated resistance to stigmatization, through building supportive communities and developing trusting relationships with HIV providers
Giles et al. (2009)	45 WLH, ages 18 to 44, living in Melbourne (Australia)	Content analysis. Data collection: Interviews (semi-structured questions). Dates: 2005 to 2006	The 15 women who had their children after their HIV diagnosis engaged in significant work including surveillance and safety work to minimize stigma and infection, information work to inform decisions and actions, accounting work to calculate risk and benefit, hope and worry work concerning a child’s infection status and impact of interventions, work to redefine an acceptable maternal identity, work to prepare an alternative story to counter the disclosure effect of the intervention and emotional work to reconcile guilt when considering these interventions
Jean et al. (2016)	19 WLH, sexually active, ages 18 to 45, living in Southern Florida (United States)	Collaborative with thematic analysis. Data collection: Interviews (open-ended questions). Date: Unknown	Decisions to conceive are influenced by women and partners; knowledge and use of safer conception practices are low. Discussion and support from partners, family and providers is limited and diminished by stigma and nondisclosure
Keegan et al. (2005)	21 WLH, ages 22 to 54, living in the United Kingdom	Interpretative phenomenological analysis. Data collection: Interviews (in depth and semi-structured). Dates: Unknown	Themes identified included: (1) difficulties with sexual functioning, specifically lowered libido and enjoyment and reduced intimacy; (2) barriers to forming new relationships: fears of HIV disclosure, fears of infecting partners; (3) coping strategies: included relationship avoidance and having casual partners to avoid disclosure; (4) safer sex: personal dislike of condoms, lack of control, lack of suitable alternatives. Women experienced a range of sexual and relationship difficulties that appear to be relatively unchanged despite the advent of HAART
Kelly et al. (2011)	6 women and 4 men living with HIV, reproductive trajectory in Northern Ireland	Qualitative narrative approach. Data collection: Interviews (in-depth) Dates: 2008 to 2009	Personal priorities and meanings are central to the negotiation of risk in sexual relationships, in which biomedical understandings of are balanced against a broader set of social expectations and desires. The need to re-negotiate a loving relationship and reproductive desires along with a desire for physical pleasure, a dislike of condoms within stable relationships and a desire to conceive without medical intervention were all given as justifications for unprotected sex in order to conceive within the context of sero-different relationship. Religious faith helps WLH embrace the uncertainties of reproduction in the context of HIV
Kelly et al. (2014)	10 women and 5 men living with HIV, different stages of disease, during reproductive trajectory in Ireland	Qualitative narrative approach. Data collection: Interviews (in-depth). Dates: 2007 to 2010	HIV positive women desire for children reflects the cultural norm of motherhood as a natural desire and a social expectation. Pregnancy signifies normality and the natural order to completing a committed relationship. The decision to become pregnant is taken against a backdrop of increased confidence in the role of treatment in lengthening lives and protecting babies from infection. Love, commitment, and desire to conceive without medical interventions, alongside the added security of an undetectable viral load, significantly impact on women’s decisions to have unprotected sex to conceive. HIV positive women are more hesitant than men to take the risk of unprotected sex with their negative partner. Achieving an undetectable viral load to protect their children from HIV infection became a major goal. Stigma continues to dominate the symbolic significance of HIV
Kirshenbaum et al. (2004)	56 women, ages 20 to 55, living in Los Angeles, Milwaukee, New York, and San Francisco (United States)	Grounded theory. Data collection: Interviews (in depth). Dates: 1998, December to 1999, August	Risk of vertical transmission was perceived by WLH but overestimated Motherhood is desired, but decision-making is impacted by beliefs about vertical transmission, strategies, stigma, religious values, attitudes of partners and health care providers, and the impact of the mother’s health and longevity on the child. When women do not want children after their diagnosis, vertical transmission risk is the main reason (but most of these women already had children). Those who become pregnant or desired children after diagnosis were more confident in the risk reduction strategies and often do not already have children
Leyva-Moral et al. (2017)	12 qualitative studies, with 50 women, published in peer-reviewed journals conducted in Brazil and the New York (United States)	Systematic review of 12 databases with meta-synthesis. Dates: 2005 to 2015. Articles assessed with Critical Appraisal Skills Programme	For pregnant WLH, pregnancy evolves as a mediated experience of commitment and dedication. The vital life experience of pregnancy is defined as an interplay of emotions, coping strategies, and feelings of satisfaction. Pregnancy in WLH is experienced and impacted by societal beliefs, as the women focuses all their efforts to take care of themselves and their babies
Leyva-Moral et al. (2018)	42 research papers, 16 with qualitative data about reproductive decisions of WLH published in peer-reviewed journals, (14 US, 1 UK, 1 Ireland)	Systematic review of qualitative and quantitative studies. Dates: 1985 to 2016. Articles assessed with Critical Appraisal Skills Programme	Socio-demographic, health status and pregnancy, religion and spirituality, beliefs and attitudes about antiretroviral therapy, clinicians, significant others, motherhood and fulfillment, fear of perinatal infection and infection of partner(s), birth control and pregnancy management are the factors that influence the reproductive decision-making process in WLH
Sanders, (2008)	9 WLH, mothers, ages 34 to 53, living in New York (United States)	Phenomenology. Data collection: Informant interviews. Dates: 2006	The experience of pregnancy for a woman with HIV is one fraught with isolation, anxiety, and distrust, but it is also one of hope for the normalcy that motherhood may bring
Sanders, (2009)	9 WLH, mothers, ages 34 to 53, living in New York (United States)	Descriptive qualitative. Data collection: Secondary data analysis to explore the lived experience of pregnancy after diagnosis with HIV (thematic analysis). Dates: 2006	Three themes: (a) unprotected sexual relations with the intent to become pregnant, (b) shifting responsibility for condom use as the relationship progressed, and (c) insufficient knowledge of how to reduce partner transmission risk in relation to childbearing. Participants were knowledgeable about the means to minimize transmission to the fetus
Siegel et al., (2006)	284 WLH, ages 20 to 45, living in New York (United States)	Qualitative content analysis. Data collection: Focused interviews of WLH. Dates: 146 interviews from 1994, October to 1996, November (prior to the advent of HAART regimens) and 138 interviews from March 2000 to April 2003 (after widespread availability of HAART)	Women in general reported a decreased sexual activity, a loss of sexual interest, and a diminished sense of sexual attractiveness following their HIV infection. The reasons for why they had discontinued sexual activity or were no longer interested in sex, included anxiety about HIV transmission, a loss of freedom and spontaneity during sex, fears of emotional hurt, not wanting the hassle of sexual relationships, a loss of sexual interest, and a diminished sense of sexual attractiveness. The types of changes in their sexuality did not differ between women in the pre-HAART and HAART eras
Toupin et al. (2019)	42 heterosexual WLH, childbearing age (mean 35 years) living in Montreal (Canada)	Qualitative study design not stated. Data collection: Semi-structured interviews. Dates: 2004 to 2005 Participants described as African (17), Haitian (12), and French Caucasian (13)	The researchers explicated the themes for decision-making of WLH at each stage of motherhood, including during conception (deciding based on the open-mindedness of providers, during pregnancy (managing transmission risks during pregnancy, making the best of medical resources, and incessant worrying about ART), and during post-partum (fearing child diagnosis, evaluating treatment during pregnancy, and reasons for continuity and change)
Walulu and Gill (2011)	15 WLH, mothers, living in Midwest (United States), > 18 years old, with at least one child living at home	Grounded theory. Data collection: Interviews (in-depth). Dates: Unknown	The core category was living for my children, which involves five areas: Knowing my diagnosis, living with HIV, taking care of myself, seeking support, and being there for my child
Wesley et al. (2000)	25 WLH, mothers, at least four months postpartum living in New Jersey (United States)	Content analysis. Data collection: Interviews (semi structured) based on Fishbein's Theory of Reasoned Action. Dates: Unknown	Motherhood is viewed as a joy and as a means of meeting unmet needs but there is a concern about children's well-being. HIV infection has a minor role in HIV-positive women's lives

*WLH* women living with HIV

From the data, 45 subthemes emerged, and these were organized into 15 themes. Then, the themes were grouped into three meta-themes to explain the factors influencing the reproductive decision-making of WLH, including: (1) Shattered identity, (2) Barriers, inequities, and misinformation, (3) Coping, resiliency, and support (see Table 3). When women learned about their HIV positive diagnosis, their meaning in life changed, and their identity altered. Yet, motherhood remained a primary goal for the women. Reproductive decision-making was perceived as a complex process influenced by many facilitators and limited by as more barriers. The facilitators helped WLH cope with their new situation to become more resilient, while the barriers made their situation more difficult to manage.

**Table 3:** Meta-themes, themes, and subthemes

Meta-themes	Themes	Sub-themes	
Shattered Identity	Womanhood	I’m shocked	
		I don’t feel like a woman anymore	
		Permanence of HIV	
		Loss of normality	
		Broken plans	
		Reduced sexual desire	
	Motherhood	Becoming mothers as a route to normality	
		Missed mothering opportunities	
		Motherhood as a social responsibility	
		Motherhood as a personal desire (value and identity)	
Barriers, Inequities, and Misinformation	System barriers	External barriers preventing access to care	
	Clinician barriers	Negative support from healthcare providers	
		They never asked me if I want to have a baby	
		Coercion and concealment	
	Inadequate information	Need for information	
		Weighing the different options	
	Individual barriers	Knowing about HIV	
		Knowing about conception methods	
		Incorrect beliefs about HIV and pregnancy	
		Uncertainty and fears	Fear of transmission
			Fear of birth defects
			Fear of leaving children alone
		Guilt	
	Family barriers	Lack of familial support	
	HIV-related stigma	Stigmatizing and stereotyping	
		Self-isolating and internalizing stigma	
		Fear to disclose, will be rejected	
	Gender-based inequalities	I just want to be loved	
		Unequal power in relationships	
	Socioeconomic barriers	Unstable situation	
Coping, Resilience, and Support	Self-care and for caring others	Treatment adherence	
		Changes in sexuality: protecting others from HIV	
		Negotiating sex	
		Family planning	Planned pregnancy
			Accidental/passive pregnancy
			Avoiding recommendations
			Child HIV + because of destiny
	Creating empowering environments		
	Humanized healthcare providers		
	Partner support		
	Hope	Medical improvement as a hope	
		Motherhood is a hope and being a mother is reason to keep fighting HIV	
		A new life: second opportunity, another chance	
	Personal Choices		
	Protection of higher power/spiritual forces		

### Shattered identity

WLH described a shattered identity, referring to two main areas: their womanhood and their motherhood experiences. When they learned of their positive status, they were devastated, and they no longer felt like a woman [61, 99]. They described how HIV assaulted their bodies; stole their sense of beauty, and left the perception of dirtiness [61]. HIV was a barrier present everywhere and all the time [61, 98–100]

“Ah, it’s just always in the bedroom, HIV. It’s always there.” [98]

"…when I looked at myself in the mirror, I could see around me the shadow of the virus (...). It was horrible because I was disgusted by myself. I looked at myself in the mirror and I could see around me, literally, a grey shadow ... And I thought “this is the virus.” [100]

Sexual and reproductive decisions were modified; their desire for intimacy reduced, sexual life changed, and breastfeeding prohibited [101]. They feared disclosing their HIV status to intimate partners, family, and friends as they lost their sense of normality [61]. Their life plans were broken when HIV unexpectedly appeared [61, 102]. Their identity was subjected to continued self-criticism that required interventions to redefine themselves. However, pregnancy was perceived to be a route to being normal and a way to feeling complete as a woman [61, 101, 103]

“It [pregnancy] is sort of like a completion of myself as a woman.” [103]

Motherhood was one of the most important social identities for women. WLH felt recognized by their community when had a child [104, 105] because motherhood is a cultural norm. The phenomenon was therefore culturally constructed [8, 104, 106]

“I just want one. I don’t want a whole house full… I just want one baby, something that’s me, a part of me. That’s something that I can develop and it’s still great too.” [106]

Some WLH felt pushed by their families and others to become mothers [107]. Many women reported losing their children to adoption, foster care, or placement with family members due to acute illnesses, chronic diseases, and/or criminal problems such as drug abuse. These broken mothering opportunities resulted in intense guilt and emotional pain. Consequently, WLH were psychologically harmed and their identity often permanently damaged [19, 108].

“When I was twenty-six, and I’m now forty-four, I didn’t see my life going that far, so I would have made different choices. I think my main thing would have been my choice of having children. I was caught in the middle of the epidemic then. I regretted not having that child. And, you know, as life went on, I still regret it.” [108]

### Barriers, inequities, and misinformation

#### Institutions and clinicians

When making reproductive decisions, WLH encountered barriers to accessing health services [106], including difficulty obtaining appointments, lengthy wait times at appointments, problems with insurance coverage, inadequate transportation, and issues related to immigration status. These barriers resulted in some women accessing services at new clinics with unfamiliar clinicians that resulted in uncomfortable feelings [106].

"When you have certain insurances, it doesn’t cover the places you want to go where you feel comfortable. You have to go outside and go to another person you don’t even have a background on. With my oldest all my prenatal care was at Hospital X; with my youngest I had a private doctor. It’s not always easy." [106]

WLH generally felt clinicians were not supportive, reporting HIV-related stigma [19, 61, 101, 102, 106, 109, 110]. The WLH felt their physicians were not helpful and explained they tried to discourage them from pregnancy due to their HIV positive status [105, 110].

“My doctor was really insensitive […]. I really felt trapped.” [110]

The physicians never asked about their desire to have children [105]. As a result they felt repressed, as their right to decide was coerced [107, 109].

"The people had pretty much brainwashed me . . . they just reared into me, telling me . . . “Right now it’s not a good time . . ., you ain’t got a place to live, you ain’t got no food, you ain’t got no job, you ain’t got this, you ain’t got that.” So, I took a look at all that and just decided to have an abortion … [I]t hurt, you know, because I wanted to keep it but I had to take a look at the situation and say, oh okay, that, that was right, yeah, okay. That’s true, though, but let me make that decision, you know, it was just like a rushed thing and . . . and I said okay, I’ll have an abortion, I can’t do anything else." [109]

WLH wanted more information about contraception methods and approaches to safe sex. They wanted to learn about their disease, including more discussions with clinicians; something they were unable to find in the health system [61, 105, 107, 111].

“I look for things that are safe 100%. There have not been recorded cases of anyone becoming infected (in oral sex), so I thought that’s 100% safe. So like a month later I was reading another one of these small leaflets and it says although the risk is small there is some risk and I started to become a bit paranoid.” [111]

#### Misinformation and fears

Their general knowledge about HIV, varied; however. WLH commonly believed they cannot be mothers. They had the wrong beliefs about the impact of HIV on their ability to become pregnant [99, 105, 106].

"Like most women, I always wanted to have a baby, but thought it wasn’t an option anymore when I was diagnosed with HIV." [105]

In terms of becoming pregnant, WLH feared the transmission risk for their child and partner, worried about possible health complications, and anticipated adverse effects from HAART [8, 19, 101, 102, 105, 107]. Yet, they still wanted to become pregnant and to give birth as naturally as possible. Although some WLH understood the transmission risk was low, they remained fearful and worried [8, 19, 101–103, 105, 107].

"My other fear is that my baby will come out positive.” [103]

In contrast, other women considered HIV to be a death sentence. They considered the psychological impact of their health and their longevity for their children when making decisions about pregnancy [8, 19, 61, 99, 102, 103].

“Am I going to get sick? Am I going to die, and the baby is going to live? I think about all of this.” [103]

WLH also confronted overwhelming guilt about their HIV negative partner, past mistakes such as drug abuse, or previous decisions to give up their children for adoption [8, 19, 61, 102, 108]. Yet again, WLH expressed their desire to change the past by becoming good mothers.

“My last daughter was also born tox-positive for cocaine... and I want to change that... I want to make sure it doesn’t happen again.” [102]

#### Stigma

Generally, WLH did not feel supported by family members as they did not want to hear about their pregnancy. As family members assumed WLH cannot have babies, the women experienced stigmatizing behaviors and comments [8, 19, 106, 109, 112].

“My mom told me to erase it out of my head... And it was always the same thing: I was selfish.” [109].

This stigma was also present in interactions with partners and clinicians [8, 19, 61, 99, 102, 105, 106, 108, 109]. As internalized stigma developed, WLH self-isolated because they feared disclosing their HIV status even to seek advice from clinicians about reproductive decisions [109]. Also, fear of rejection made WLH especially reluctant to disclose their HIV status to partners and friends when speaking about pregnancy [8, 61, 99, 105–108].

"Stigma …makes that person not want to talk about it [pregnancy]. So, you have already labeled me, you already said how you feel about it. If we are talking, and you already said something bad about someone who has the virus -- why would I open up to you? You’re going to talk about me. And see my feelings are going to get hurt.” [106]

#### Feeling undermined

WLH want to be loved and to have an intimate partner [61]. But occasionally their decisions were coerced by men, who forced WLH to have unprotected sex or to become pregnant [61, 106]. When men knew they were HIV positive or when they became pregnant, the relationship was disrupted. In these cases, some woman felt abandoned or, on the contrary, others had subsequent unwanted pregnancies [61].

"I was always the one pushing him to use condoms, and he didn’t want to. Eventually when we were living together, I just stopped. It’s not always going to be my responsibility to push that." [112]

Finally, WLH sometimes experienced homelessness, poverty, domestic violence, drug abuse, and other marginalizing situations. Instability in their lives made reproductive decisions difficult to manage, including whether or not to continue with unplanned pregnancies [102, 109]. Some women believed having a child without the resources to create a stable family environment was irresponsible [102].

“It’s not an irresponsible thing to have a child. It’s an irresponsible thing to have a child without a father, without a decent income, without a place to live and without the ability to take care of this child…while dealing with your own stuff.” [102]

### Coping, resiliency, and support

Sexuality and reproduction overlap with love, intimacy, and commitment but all are disturbed by HIV. As the WLH did not want to harm others by transmitting their HIV, they searched for approaches for safe sex or chose celibacy [61]. WLH reported barriers to using contraceptives such as cost and rejection by their partner [104]. The women needed strategies to negotiate safe sex [61, 104]. This process was described as passive negotiation in studies [61, 111].

“I just told them it was because you don’t know where I come from, I don’t know where you come from, so it is good to be careful and at the same time I don’t want to get pregnant.” [111]

WLH considered pregnancy planning to protect their health, as well as their partner, prior to trying to conceive [106]. Nonetheless, some women believed reproductive decisions were passive plans, even accidental situations; and they needed to assume responsibility for the consequences [102, 106]. Some WLH reported lacking confidence in HAART to reduce the risk of mother-to-child transmission [107]. They believed that if the infant is born infected this is destiny [102].

“It’s not really a planning thing, they just talk about it if they want to have a baby, and if it happens then it happens.” [106]

Other WLH preferred to conceive without medical intervention so they engaged in unprotected sex. For these women, love and commitment with their partners was more important than the risk because childbirth should not be medicalized [105, 106].

“When you find a guy and you feel comfortable with them and they accept what is going on with you, you cannot use condoms, even though you know you can get re-infected…when you are blinded by that person you take risks, I take risks, and say ain’t nothing going to happen to me, ain’t nothing happened to me so far so what the hell.” [106]

Sharing their status with others was a coping strategy identified by WLH to accept their diagnosis. Creating empowering environments such as support groups for people to understand their situation appeared to be helpful [109].

“Surrounding myself around people that’s HIV… It helps me a lot, it chills me down.” [109]

However, intimate partners were the most important person for reproductive decision-making as they offered the women the self-confidence they needed to make a decision [101, 105, 106]. In some cases, WLH reported their relationships were stronger following HIV diagnosis due to the increased partner support [101].

"He’s [partner] with me, he’s got my back 110%. So, anything I decide I want to do he supports me -- there’s not a lot of men that do that. When I met and let him know what my status was, he told me “and what?” He didn’t see my status he saw that person that I was, that I am, he knows that I am a good person and that’s what." [106]

Despite the challenges when seeking advice about pregnancy from clinicians and family, the women wanted to make a good decision. They were not discouraged from wanting children by negative input from family members or their clinicians [106, 110].

"I really don’t care for…what family has to say, because …as a grown individual you have to be grown enough… to make your own decisions." [106]

“Being a mother makes me grow on a personal level, but the doctor doesn’t think of motherhood as medicine for the soul, a morale booster that affects me psychologically.” [110]

However, WLH shared their decision with God as they believed their situation was the plan God had for them [8, 19, 61, 99, 102, 108, 110]. God plays an essential role in reproductive decision-making as ‘He’ will protect their children, so the final decision is shared with ‘Him.’ [99] WLH felt relief because they could depend on spiritual forces, including the greater protection from God's power.

"But basically, it’s a decision I made with my higher power. I just ask God to show me what to do." [19]

“And if I take care of myself, I think maybe God will give me a lot more time of life. Because I pray to God everyday: “God, give me life, I want see my daughters to grow up with me, I want them to see me well, I want myself being able to work" (Alvarez-del Arco et al., 2018).

When WLH were aware of medical advancements related to pregnancy, the possibility of becoming a mother was real [105]. Mothering gave them a reason to continue their fight against HIV as they wanted to live, and they could be stronger [8, 19, 61, 99]. Moreover, some WLH felt they received a second chance, as they had an opportunity to correct their past parenting mistakes and to capture their missed mothering opportunities [19, 108].

"However, over the years, with improvements in treatment and people with HIV living longer, it started to feel possible. The doctors told us that the risk of the baby having HIV has gone down to 1%" [105].

## Discussion

Once a woman is diagnosed with HIV, life changes and identity alters. Many WLH cancel motherhood plans due to knowledge deficits, misinformation, stigma, and judgment. Being a mother is defined by their feminine identity as well as social expectations defined by the cultural context. As such, WLH seek to salvage their role as "good mothers" to stabilize their identity and to maintain their social value threatened by HIV [6]. In general, people living with HIV experience a wide range of negative emotions such as stress [113], fear [114], guilt [107], hopelessness [115], and internal stigma [116]. In the case of WLH engaged in reproductive decision-making following broken mothering opportunities, their guilt and pain is elevated.

In consonance with available evidence, this study found a lack of knowledge about the relationship between living with HIV, sexual health, and reproductive decision-making [21, 117]. The women believed they should not become mothers, and their reproductive desires were broken by the force of this belief [117]. As Fransen and Guarinieri [24] concluded, a woman´s desire to have children is not influenced by the HIV diagnosis, but their ability to act upon this desire is blocked by the stigma resulting from the negative attitudes of people in their lives. Moreover, their sexuality is modified, and the feeling of normality is lost. In this regard, WLH experience sexual problems including decreased function and diminished desire, activity, and satisfaction compared to uninfected women [118].

The WLH wanted the support of their clinicians as they tried to understand the possibility of safely having a child [99]. Their role is essential in correcting the perception of risk in helping WLH make an informed decision about safe conception and pregnancy [24, 117]. However, some studies suggested there is a persistent stigma related to pregnancy in HIV-affected couples. In this case, clinicians are more likely to ask their patients about contraception practices rather than reproductive intentions [20]. But, WLH want to be viewed as a whole person, not only as a diseased entity [18]. For these reasons, WLH perceive clinician assumptions about their sexual practices and reproductive goals are barriers to reproductive discussions. Lassi et al. [119] recommended clinicians engage in preconception counseling for women of reproductive age when they test HIV-positive as well as screening with their partners prior to pregnancy. Comprehensive reproductive health counseling for WLH is necessary, but clinicians need to be taught how to be authentically present and to be engaged in shared decision-making with HIV-affected couples [20].

From this qualitative synthesis, stigma was evidenced in the interactions WLH reported with friends, families, partners, and clinicians [20, 26]. Stigma expands into marginalization, stress, uncertainty, and isolation [8]. The WLH experience insecurity about their prognoses, potential treatments, changing social relationships, and new personal identity [120]. Significant stigma for WLH results from barriers to accessing preventive services and health care [121]. WLH have similar concerns about clinician support and uncertainty about health care as people living with acute and chronic conditions.

Reproductive decision-making for each WLH is unique, as they use their repertoire of coping mechanisms to stay alive. When entering a support group for example, they can become part of a larger reality with peer support that provides collective strength [21]. Also, partner support is indispensable as they are identified as the most important person in reproductive decision-making [10]. Although clinicians are often not actively involved in reproductive decision-making, clinical advancements offer another opportunity for the women to correct past parenting mistakes as hope to become a good mother. Hope is capable of changing their lives and motivating the women to go forward [122] in fighting the HIV.

Generally, WLH wanted to make their own reproductive decisions despite many barriers while recognizing they needed more support. The women frequently noted spiritual forces protected them, as well as planned their lives. As such, these women felt a strong duty to care for their babies in a way that facilitated self-care. The risk of transmitting their infection to their fetus, or their partner, is their most pressing concern [6, 18]. They also fear death because they do not want to abandon their children [123]. Spirituality becomes an effective coping mechanism for WLH to provide meaning, guidance, support, protection, and inner strength [124–127].

### Study limitations

There are several limitations to consider. First, the synthesis relied on the analyzed data from each article that might not reflect the meaning from the raw data. Second, to synthesize studies from countries with comparable social wellbeing and economic conditions, the search was limited to OECD member countries. As such, the review may not be generalizable to developing countries. In addition, almost half the included studies were conducted in urban areas of the United States which may further limit the generalizability of the findings within the target countries. Despite these limitations, the search included multiple languages due to the multinational team, extensive database searches, and comparable environments. Third, the studies spanned almost three decades, defined by the period of HAART. Despite the wide span, there was data consistency across the decades. Fourth, the systematic review was completed from 1995 to 2018 with an update in 2019 introducing an increased possibility for search bias. However, the broad search strategy minimized the impact of this concern. Finally, the search might have excluded studies not published in non-indexed journals or in different contexts, such as thesis or dissertations. However, the search strategy and the database selection were based on published recommended practices.

## Conclusion

For WLH, reproductive decision-making is a complex process based upon incomplete information and inaccurate perspectives. In developed countries, WLH engage in reproductive decision-making more frequently due to advances in pharmaceuticals and better evidence-based practices while receiving limited assistance from family, friends, partners, and clinicians. Decisions regarding reproduction are individual but women are not able to make an informed decision without guidance and medical consultation. Clinicians need to be authentically present for discussions and open to the new possibilities for WLH in the context of the modern person-centered health system. An integrated approach with comprehensive multidisciplinary counseling is necessary to address reproductive decision-making and sexual health as a right. Clinicians need to initiate discussions for WLH in the same way as other women.

In clinical practice, WLH should be engaged with a human caring approach to identify personal preferences, consider individual situations, and provide person-centered care. Clinicians should learn to collaborate with WLH in reproductive decision-making through regular family planning conversations and routine sexual health assessments. For more effective relationships with WLH, clinicians need to be authentically present, including demonstrating compassion and understanding to minimize the stigma associated with sexual health. Finally, evidence-based practice guidelines for sexual health and family planning need to be developed for WLH to consistently deliver culturally competent, integrated, and holistic person-centered care.

## Data Availability

The data used and/or analyzed during the current study are available from the corresponding author on reasonable request.

## Abbreviations

CASP: Critical Appraisal Skills Programme
ENTREQ: Enhancing transparency in reporting the synthesis of qualitative research
HAART: Highly active antiretroviral therapy
HIV: Human immunodeficiency virus
JBI: Joanna Briggs Institute
OECD: Organisation for Economic Cooperation and Development
PRISMA: Preferred reporting items for systematic reviews and meta-analyses
PROSPERO: International prospective register of systematic reviews
QRS: Qualitative research synthesis
WLH: Women living with HIV
